# Urétérocèle sur uretère simplex chez l’enfant: aspects cliniques et thérapeutiques

**DOI:** 10.11604/pamj.2021.38.345.15142

**Published:** 2021-04-09

**Authors:** Samia Belhassen, Aziza Elezzi, Saida Hidouri, Rachida Laamiri, Sana Mosbahi, Amine Ksiaa, Lassad Sahnoun, Mongi Mekki, Mohsen Belguith, Abdellatif Nouri

**Affiliations:** 1 Service de Chirurgie Pédiatrique, Hôpital Fattouma Bourguiba de Monastir, Monastir, Tunisie,; 2 Laboratoire de Recherche des Pathologies Malformatives et Tumorales LR12SP13, Centre Hospitalo-universitaire Fattouma-Bourguiba de Monastir, Monastir, Tunisie

**Keywords:** Urétérocèle, système simple, enfant, uropathie, Ureterocele, simple system, child, uropathy

## Abstract

L´urétérocèle est une dilatation pseudo-kystique de l´uretère terminal sous muqueux. C´est une uropathie malformative rare surtout si elle survient sur un uretère simplex. Il s´agit d´une étude rétrospective menée sur dix ans, de 12 dossiers de malades colligés au Service de Chirurgie Pédiatrique de l´Hôpital Fattouma Bourguiba de Monastir entre 2006 et 2016. L´âge moyen de nos malades est de 2,7 ans avec des limites allant de 7 jours à 11 ans, le sex-ratio est de 1. Le tableau clinique a été dominé par la fièvre en rapport avec une infection urinaire haute. Le diagnostic a été posé essentiellement par l´échographie rénale et vésicale, l´urographie intraveineuse (UIV) et l´uréthro-cystographie rétrograde (UCR). L´urétérocèle était unilatéral dans 10 cas et bilatérale dans 2 cas soit un total de 14 cas d´urétérocèles. Tous ont été sur uretère simplex et ont été opérées par voie endoscopique. Aucun incident peropératoire n´a été noté. Les suites opératoires étaient simples. Une amélioration clinique et radiologique a été obtenue dans tous les cas. L´urétérocèle sur uretère simplex est une malformation urinaire très rare. Non diagnostiquée à temps, elle peut retentir sur le haut appareil. Le traitement endoscopique est une alternative séduisante avec des résultats satisfaisants.

## Abstract (en)

Ureterocele is a pseudo-cyst dilation of the terminal submucosal ureter. It is a rare malformative uropathy, in particular associated with simplex ureter. We conducted a retrospective study over a period of 10 years. Twelve medical records of patients whose data were collected at the Department of Paediatric Surgery of Monastir between 2006 and 2016 were examined. The average age of patients was 2.7 years (ranging from 7 days to 11 years) with a sex ratio of 1. Patients´ clinical status was dominated by fever due to upper urinary tract infection. Diagnosis was mainly based on renal bladder ultrasound, intravenous urography (IVU) and retrograde urethrography and cystography. Ureterocele was unilateral in 10 cases and bilateral in 2 cases (on a total of 14 cases). It was associated with simplex ureter in all cases and all patients underwent endoscopic surgery. No perioperative adverse event was reported. The postoperative course was uneventful. Clinical and radiological improvements were reported in all cases. Ureterocele associated with simplex ureter is a very rare urinary abnormality. Early diagnosis is essential to avoid upper urinary tract involvement. Endoscopic treatment is a good alternative leading to satisfactory results.

## Introduction

L´urétérocèle est une malformation urinaire congénitale rare surtout si elle survient sur un uretère simplex. Elle est caractérisée par une dilatation pseudo-kystique de la portion terminale de l´uretère. L´avènement de l´échographie anténatale a rendu le diagnostic de cette affection de plus en plus précoce. Sa symptomatologie est variable. Quant au traitement, la voie endoscopique est de plus en plus utilisée. Le but de ce travail est de discuter les différents aspects cliniques, paracliniques et thérapeutiques de l´urétérocèle sur uretère simplex chez l´enfant.

## Méthodes

Il s´agit d´une étude rétrospective sur 10 ans (2006-2016) au Service de Chirurgie Pédiatrique de l´Hôpital Fattouma Bourguiba de Monastir. L´étude s´est intéressée aux aspects cliniques, paracliniques et thérapeutiques ainsi que l´évolution après le traitement endoscopique de cette affection. L´âge de la consultation, la symptomatologie initiale, la biologie, les explorations radiologiques, le traitement et l´évolution ont été recueillis à partir des dossiers médicaux et des comptes rendus opératoires.

## Résultats

**Figure 1:**
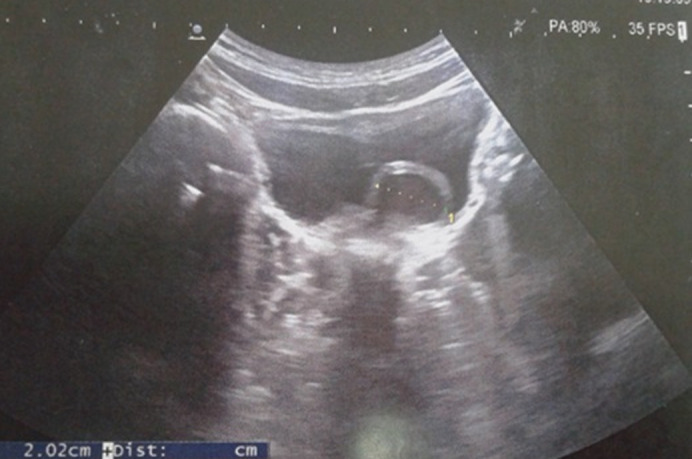
échographie vésicale; aspect d´urétérocèle

**Figure 2:**
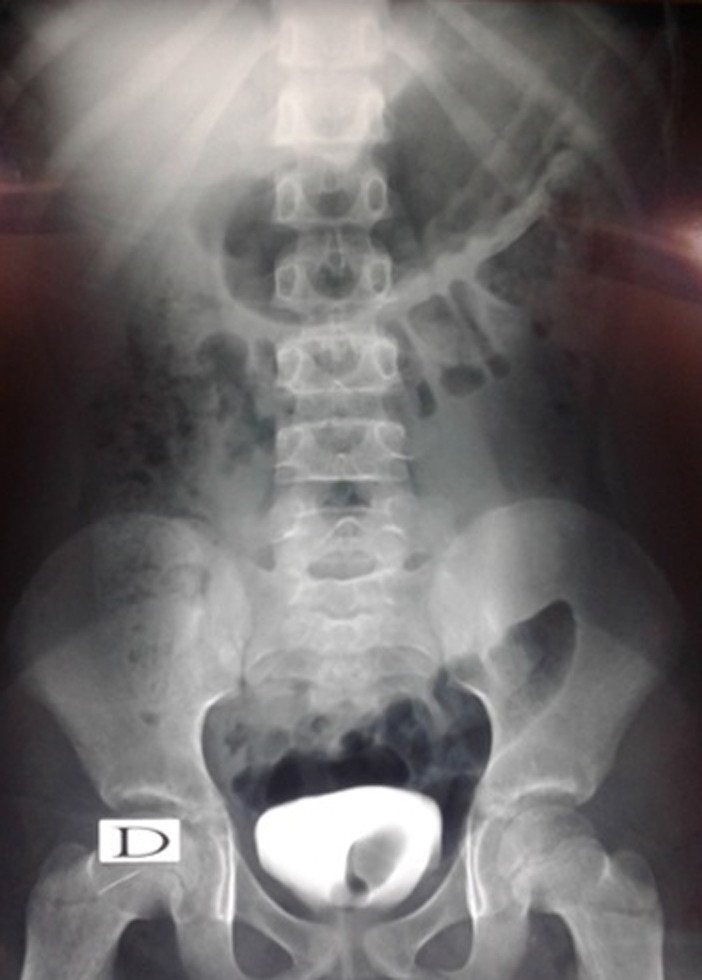
UCR; urétérocèle isolé

**Figure 3:**
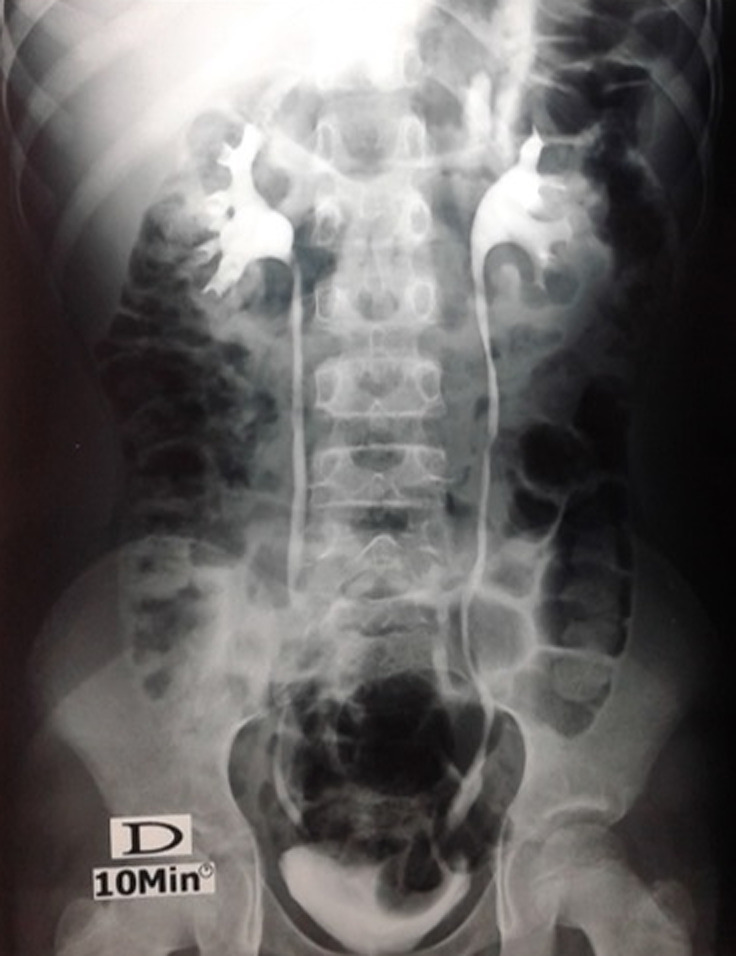
UIV; urétérocèle gauche

**Figure 4:**
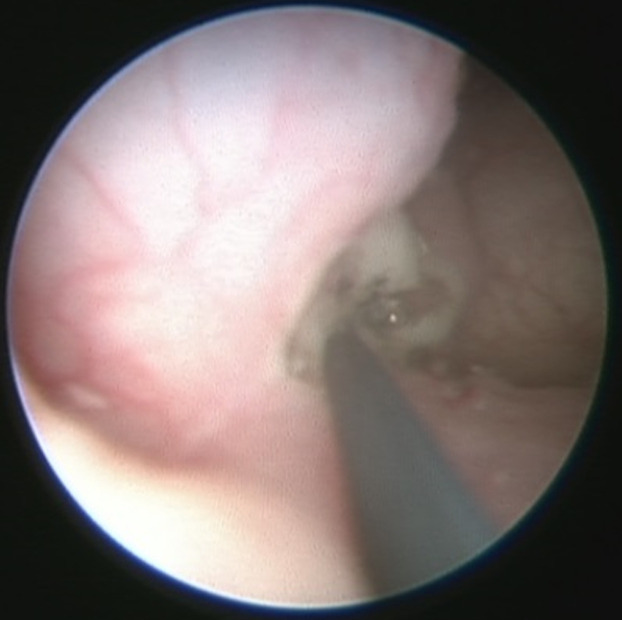
ponction endoscopique de l´urétérocèle

Cette étude concernait 12 enfants dont 6 garçons et 6 filles, pour un sex-ratio = 1. L´âge moyen de nos malades était de 2,7 ans. La circonstance de découverte était anténatale dans un cas, une infection urinaire haute dans 7 cas. Une hématurie a été révélatrice dans 2 cas, des douleurs lombaires dans 2 cas. Le diagnostic a été posé à l´échographie rénale et vésicale montrant la classique image de kyste intravésical dans 10 cas (Figure 1). L´UCR pratiqué chez 9 malades était normale dans 6 cas, visualisant l´urétérocèle dans 3 cas (Figure 2). L´UIV pratiquée chez 10 malades a visualisé l´urétérocèle dans 8 cas (Figure 3). L´urétérocèle a était à droite dans 8 cas, à gauche dans 2 cas et bilatérale dans 2 cas soit un total de 14 urétérocèles. Le retentissement sur le haut appareil a été marqué par une dilatation urétéro-pyélo-calicielle (UPC) dans un cas. La fonction rénale était correcte chez tous les enfants. Tous nos malades ont eu un traitement chirurgical par voie endoscopique (Figure 4). Toutes ces urétérocèles étaient intravésicales et leurs tailles variaient entre 6 et 20 mm. Le geste opératoire a consisté à faire une incision semi-circulaire à la base de l´urétérocèle. La durée moyenne de l´acte était de 30min sans incident, sans drainage vésical. Les suites opératoires étaient simples. La sortie a été faite au bout de 24h. L´évolution était bonne et sans complications. Le contrôle échographique n´a pas montré de récidive de l´urétérocèle dans tous les cas et une régression de la dilatation urétéro-pyélocalicielle préexistante dans le cas déjà cité. Aucune autre exploration radiologique n´a été jugée nécessaire devant l´absence de symptomatologie clinique postopératoire. Le recul moyen est de 6 ans 8 mois.

## Discussion

L´urétérocèle est une malformation rare dont la fréquence est estimée à 1/4000 naissance [1]. Elle se définit comme étant une dilatation pseudo-kystique du segment sous muqueux de l´uretère intravésical entre le hiatus du détrusor et le méat urétéral [2]. L´urétérocèle est plus fréquente chez la fille que chez le garçon. Dans notre série le sex-ratio est de 1. Une prédominance de l´urétérocèle à droite est notée. Selon les séries publiées, il n´y a pas de prédilection de côté [3]. La symptomatologie est variée et dominée par l´infection urinaire. Dans notre série la fièvre en rapport avec l´infection urinaire était présente dans 59% des cas. L´hématurie isolée peut être révélatrice de l´urétérocèle ainsi que la lithiase. L´échographie permet le diagnostic dans la plupart des cas. Dans notre série, l´échographie prénatale a permis le diagnostic dans un cas ce qui permet le traitement précoce de l´urétérocèle avant même l´apparition des complications [4].

L'UIV met en évidence l´image typique de l´urétérocèle en tête de serpent tout en écartant la présence d´un système double. La cystoscopie met en évidence cette urétérocèle en la visualisant directement. Selon Coplen et Duckett [5], le traitement endoscopique doit être indiqué de première intention. En effet l´incision de l´urétérocèle permet la décompression de l´urétérocèle et des voies urinaires. Plusieurs auteurs, Scovell *et al*. [6], Sental *et al*. [7] et Westesson et Goldman [8] ont montré l´efficacité du traitement endoscopique; cette technique permet de traiter définitivement 93% des urétérocèles intra-vésicales selon Blyth *et al*. [9]. Dans une série de Barett *et al*. [10], cette technique a permis une amélioration de la fonction rénale dans 95%. Ces résultats sont améliorés par la mise en place de sonde « double J » afin de prévenir les sténoses postopératoires et les récidives [11]. Toutefois on doit veiller au risque d´apparition d´un reflux vésico-urétéral par une surveillance radiologique [12]. Ce qui n´a pas été fait dans notre série devant l´absence de symptomatologie clinique.

## Conclusion

L´urétérocèle sur uretère simplex est affection rare chez l´enfant. Elle est diagnostiquée au décours d´une infection urinaire dans la plupart des cas. Le traitement endoscopique est une technique mini invasive facile, reproductible et efficace avec un bon résultat à long terme.

### Etat des connaissances sur le sujet

- L´urétérocèle est une pathologie rare surtout sur uretère simplex;
- Le diagnostic anténatal est difficile;
- Son traitement se fait de plus en plus par voie endoscopique.

### Contribution de notre étude à la connaissance

- Le nombre relativement important par rapport à la rareté de cette pathologie et essentiellement sur uretère simplex;
- Le traitement de tous les malades a été fait par voie endoscopique d´où l´intérêt de cette voie actuellement;
- L´évolution satisfaisante de nos malades et l´intérêt d´une surveillance à long terme de ces malades.
